# The transcription factor GCN4 contributes to maintaining intracellular amino acid contents under nitrogen-limiting conditions in the mushroom *Ganoderma lucidum*

**DOI:** 10.1186/s12934-023-02213-z

**Published:** 2023-10-10

**Authors:** Lingdan Lian, Jinjin Qiao, Xiaoyu Guo, Zhenzhen Xing, Ang Ren, Mingwen Zhao, Jing Zhu

**Affiliations:** 1 Key Laboratory of Agricultural Environmental Microbiology, Microbiology Department, College of Life Sciences, Ministry of Agriculture and Rural Affairs, Nanjing Agricultural University, Jiangsu, Nanjing, 210095 PR China; 2 Department of Bioengineering, College of Food Science, South China Agricultural University, Guangzhou, 510642 PR China; 3 College of Life Sciences, Nanjing Agricultural University, No.1 Weigang, Nanjing, Jiangsu, 210095 PR China

**Keywords:** GCN4, Amino acids, Nitrogen, *Ganoderma lucidum*

## Abstract

**Background:**

Edible mushrooms are delicious in flavour and rich in high-quality protein and amino acids required by humans. A transcription factor, general control nonderepressible 4 (GCN4), can regulate the expression of genes involved in amino acid metabolism in yeast and mammals. A previous study revealed that GCN4 plays a pivotal role in nitrogen utilization and growth in *Ganoderma lucidum*. However, its regulation is nearly unknown in mushrooms.

**Results:**

In this study, we found that the amino acid contents reached 120.51 mg per gram of mycelia in the WT strain under 60 mM asparagine (Asn) conditions, but decreased by 62.96% under 3 mM Asn conditions. Second, silencing of *gcn4* resulted in a 54.2% decrease in amino acid contents under 60 mM Asn, especially for the essential and monosodium glutamate-like flavour amino acids. However, these effects were more pronounced under 3 mM Asn. Third, silencing of *gcn4* markedly inhibited the expression of amino acid biosynthesis and transport genes. In addition, GCN4 enhanced the tricarboxylic acid cycle (TCA) and glycolytic pathway and inhibited the activity of target of rapamycin complex 1 (TORC1), thus being beneficial for maintaining amino acid homeostasis.

**Conclusion:**

This study confirmed that GCN4 contributes to maintaining the amino acid contents in mushrooms under low concentrations of nitrogen. In conclusion, our study provides a research basis for GCN4 to regulate amino acid synthesis and improve the nutrient contents of edible mushrooms.

**Supplementary Information:**

The online version contains supplementary material available at 10.1186/s12934-023-02213-z.

## Background

Edible mushrooms are considered a so-called ‘superfood’ or functional food due to their umami flavour and the presence of triterpenoids, polysaccharides, proteins, peptides, and phenolic compounds [1]. Proteins in edible mushrooms are rich and the content is as high as 18.87–36.96% of the dry weight, with a complete profile of essential amino acids (EAAs) required for adults [2]. If fungal protein is obtained by fermentation, it can replace 20% of global beef consumption and reduce deforestation and carbon dioxide emissions by 56% [3]. Plant proteins do not meet human dietetic requirements due to the lack of some essential amino acids. Therefore, mushroom proteins have the potential to serve as a viable supplement to animal and plant proteins [4].

Amino acid composition, especially the content of essential amino acids is a reliable indicator of protein quality [2]. Edible fungal amino acids not only contain the complete amino acids profile but also have unique flavours and functions [5–7]. The content of essential amino acids in *Calvatia gigantea* accounts for more than 56% of the content of free amino acids [8]. As an excellent source of the essential amino acids, L-lysine content is high in *Flammulina velutipes* [9]. The amino acids in mushrooms are divided into four groups: the umami taste amino acids, the sweet amino acids, the bitter amino acids and the tasteless amino acids [5, 10]. Among them, aspartic acid and glutamic acid, belonging to the monosodium glutamate-like (MSG-like) component class, are relatively abundant in most mushrooms, and provide the umami or palatable flavour of mushrooms [11, 12]. Therefore, the content and composition of amino acids are important indicators to evaluate the nutrition and flavor of edible mushrooms.

In edible mushrooms, changes in environmental or cultivation conditions, as well as the expression of some key genes involved in amino acid synthesis, affect the amino acid contents. Using the wheat stalk as the growth substrate led to good essential amino acid patterns in *Pleurotus ostreatus* [13, 14]. High sawdust content or a high C/N value were beneficial to high levels of protein and amino acids in *P. eryngii* [15]. Blue light and chitosan treatment increased the content of lysine in *F. velutipes* [16]. In addition, genes involved in the biosynthesis of specific critical amino acids, such as lysine in *F. velutipes* and cysteine in *Lentinus edodes*, were studied. Overexpression of *sdh*, the last step of fungal lysine biosynthesis, significantly increased the lysine content in *F. velutipes* [17]. The formation of unique aromas in *L. edodes* is related to the synthesis of cysteine. A cysteine synthesis gene, cysteine sulfoxide lyase gene, has been found to affect the formation of organosulfur compounds in *L. edodes* [18]. Although these studies have focused on the amino acid biosynthesis in edible mushrooms, there are few investigations on the regulation mechanism of amino acid contents.

Organisms can maintain the intracellular amino acid homeostasis in many aspects, including regulating the activities of amino acid transporters, de novo synthesis and degradation of proteins, in which signals, genes and pathways participate [19]. Gcn4, a basic leucine zipper (bZIP) transcription factor, is the master transcriptional activator of genes for amino acid biosynthesis in fungi [20, 21]. Analysis of cDNA microarray expression profiles in *Saccharomyces cerevisiae* revealed that GCN4 regulates the expression of more than 30 amino acid biosynthesis genes [22, 23]. CpcA (homologue of GCN4) in *Aspergillus fumigatus* was the transcriptional activator of the cross-pathway control (CPC) system of amino acid biosynthesis [24]. In addition, the tryptophan synthase-encoding gene (*trpB*) and arginine synthase-encoding gene (*argB*) in *A. nidulans* were regulated by CpcA [25, 26]. However, the functions of GCN4 have rarely been reported in basidiomycetes, especially in edible mushrooms.

As a representative edible and medicinal mushroom, *Ganoderma lucidum* has attracted extensive attention and research interest due to its important pharmacological and high economic value [27], the complete genome sequence information [28] and the well-establishment of the genetic transformation system [29]. *G. lucidum* is susceptible to a low nitrogen during the late growth and development stages. In our previous study, we found that GCN4 was activated to maintain growth under the low-nitrogen conditions [30]. Here we found that GCN4 was important for the intracellular amino acid homeostasis, especially the MSG-like taste amino acid and the essential amino acid contents. Furthermore, GCN4 promoted the expression of the genes involved in the amino acid homeostasis under the low concentrations of nitrogen. In addition, GCN4 increased the TCA cycle and glycolysis pathway to provide precursors for amino acid biosynthesis, and inhibited the activity of target of rapamycin complex 1 (TORC1) to reduce the utilization of amino acids. In conclusion, our findings provide a reference for the exploration of intracellular amino acid regulatory networks in edible mushrooms.

## Results

### **The amino acid contents of *Ganoderma lucidum*under low-nitrogen concentrations**

**Fig. 1:**
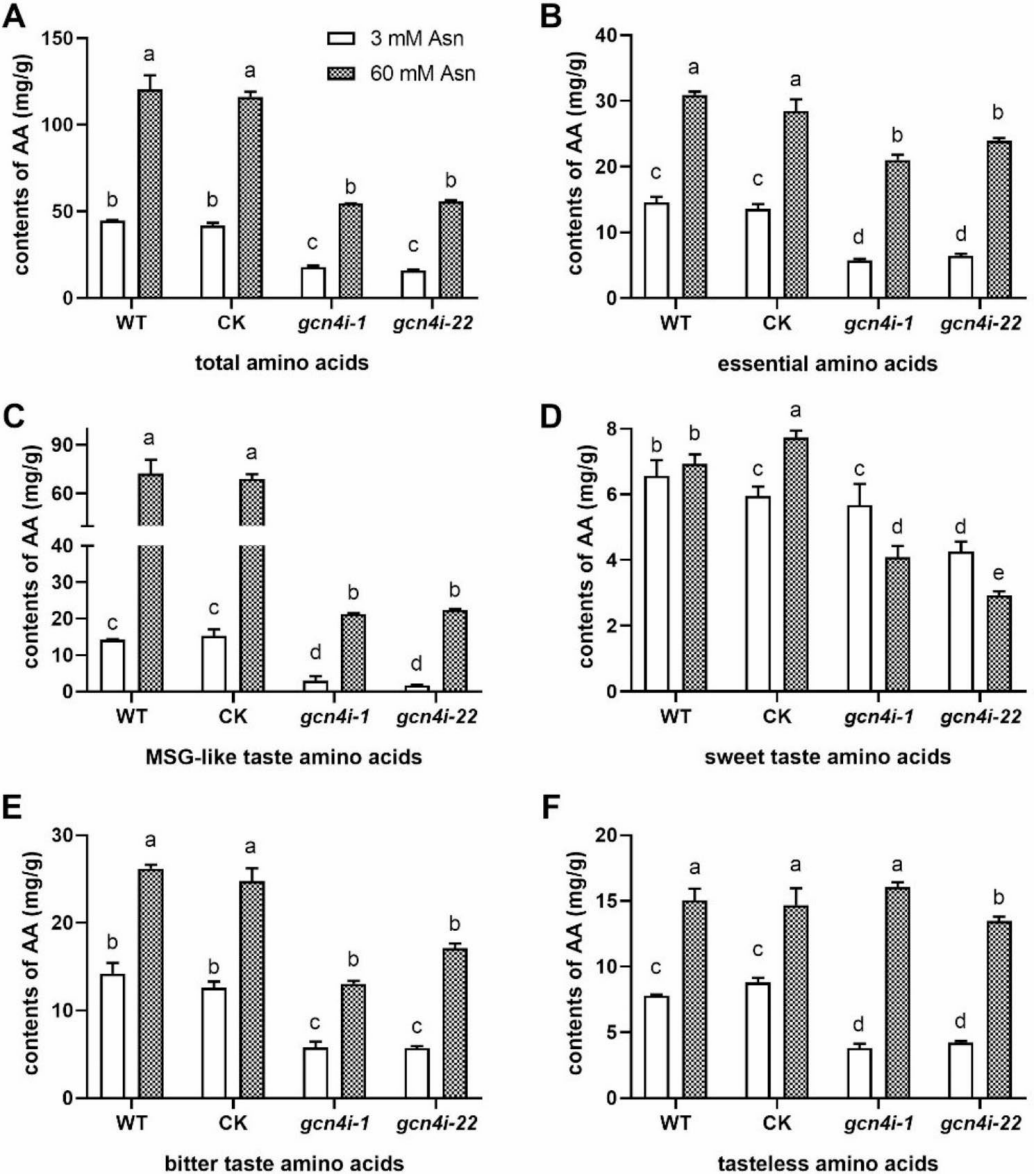
Involvement of GCN4 in regulating intracellular amino acid contents. Intracellular amino acid contents **(A)**, essential amino acid contents **(B)**, MSG-like flavour amino acid contents **(C)**, sweet flavour amino acid contents **(D)**, bitter flavour amino acid contents **(E)** and flavourless amino acid contents **(F)** in WT and *gcn4*-silenced strains. MSG-like flavour amino acids include aspartic acid and glutamic acid. Sweet flavour amino acids include threonine, serine, glycine and alanine. Bitter flavour amino acids include valine, methionine, isoleucine, leucine, phenylalanine, histidine, arginine and tryptophan. Flavourless amino acids include cystine, tyrosine and lysine. The data are presented as the mean ± SD (n = 3). Statistical significance is represented by different letters corresponding to *P* < 0.05 based on Tukey′s test

Nitrogen is the main macronutrient for fungal structure and energy requirements. *G. lucidum* suffers nitrogen-limiting conditions during its growth. To determine the specific mechanism, we screened for dominant nitrogen sources for the growth of *G. lucidum* mycelia. The *G. lucidum* had good growth status when asparagine (Asn) was used as the sole nitrogen source (Fig. S1). Therefore, low concentrations of Asn were utilized to mimic nitrogen-limiting conditions and explored the intracellular amino acid content. The content of each detected amino acid decreased under low-nitrogen conditions (Table 1), compared with that under the high-nitrogen conditions, except for threonine. As shown in Fig. 1A, the amino acid content reached 120.51 mg/g in the WT strain under the 60 mM Asn condition. However, the content of amino acids under the 3 mM Asn decreased by 62.96% compared with that under 60 mM Asn (Fig. 1A). Under the low concentration of nitrogen, the contents of essential amino acids decreased by 52.63% compared with the high concentration of nitrogen (Fig. 1B). The contents of MSG-like, sweet, bitter and tasteless amino acids under 3 mM Asn decreased by 79.06%, 14.65%, 47.35% and 44.23%, respectively, compared with those under 60 mM Asn (Fig. 1C-F). Taken together, these results showed that a high concentration of nitrogen benefits the amino acid accumulation, while a low concentration of nitrogen resulted in a decrease in amino acid contents in *G. lucidum*.

**Table 1:** The amino acid contents of WT and *gcn4*-silenced strains under different nitrogen conditions

	3mM Asn WT	60 mM Asn WT	3 mM Asn SiControl	60 mM Asn SiControl	3 mM Asn *gcn4i-1*	60 mM Asn *gcn4i-1*	3 mM Asn *gcn4i-22*	60 mM Asn *gcn4i-22*
Asp	8.86 ± 1.03^e^	61.4 ± 8.83^a^	11.1 ± 1.15^d^	54.31 ± 2.49^b^	1.64 ± 1.24^de^	17.71 ± 0.38^c^	0.59 ± 0.04^f^	17.81 ± 0.14^c^
Glu	5.42 ± 0.98^c^	10.95 ± 0.49^b^	4.22 ± 0.65^ cd^	14.67 ± 1.42^a^	1.34 ± 0.13^e^	3.67 ± 0.4^d^	1.13 ± 0.1^e^	4.43 ± 0.44^ cd^
**Ser**	**0.43 ± 0.09** ^**bc**^	**0.7 ± 0.17** ^**a**^	**0.55 ± 0.07** ^**ab**^	**0.69 ± 0.07** ^**a**^	**0.44 ± 0.05** ^**bc**^	**0.64 ± 0.1** ^**a**^	**0.37 ± 0.02** ^**c**^	**0.43 ± 0.02** ^**bc**^
**His**	**2.27 ± 0.71** ^**b**^	**3.22 ± 0.11** ^**a**^	**1.47 ± 0.49** ^**c**^	**2.99 ± 0.51** ^**a**^	**0.51 ± 0.12** ^**d**^	**1.08 ± 0.26** ^** cd**^	**0.48 ± 0.02** ^**d**^	**0.59 ± 0.05** ^**d**^
**Gly**	**1.1 ± 0.06** ^** cd**^	**1.35 ± 0.06** ^**c**^	**1.11 ± 0.19** ^** cd**^	**2.64 ± 0.08** ^**a**^	**2.08 ± 0.39** ^**b**^	**0.88 ± 0.14** ^**d**^	**2.18 ± 0.22** ^**b**^	**0.85 ± 0.1** ^**d**^
**Thr**	**2.23 ± 0.13** ^**a**^	**1.27 ± 0.09** ^**c**^	**1.78 ± 0.05** ^**b**^	**0.95 ± 0.06** ^**d**^	**1.14 ± 0.21** ^** cd**^	**1.27 ± 0.28** ^**c**^	**0.42 ± 0.17** ^**e**^	**0.98 ± 0.09** ^**d**^
**Arg**	**2.6 ± 0.34** ^**a**^	**2.68 ± 0.32** ^**a**^	**2.97 ± 0.35** ^**a**^	**2.69 ± 0.09** ^**a**^	**1.1 ± 0.12** ^**d**^	**2.06 ± 0.11** ^**b**^	**0.82 ± 0.06** ^**d**^	**1.61 ± 0.1** ^**c**^
**Ala**	**2.82 ± 0.48** ^**b**^	**3.62 ± 0.24** ^**a**^	**2.52 ± 0.11** ^**b**^	**3.45 ± 0.23** ^**a**^	**2.01 ± 0.29** ^**c**^	**1.26 ± 0.14** ^**d**^	**1.29 ± 0.23** ^**d**^	**0.67 ± 0.04** ^**e**^
**Tyr**	**2.42 ± 0.28** ^**d**^	**3.09 ± 0.46** ^**bc**^	**2.54 ± 0.29** ^** cd**^	**3.18 ± 0.25** ^**b**^	**1.76 ± 0.53** ^**e**^	**4.15 ± 0.11** ^**a**^	**1.3 ± 0.24** ^**e**^	**3.44 ± 0.19** ^**b**^
**Cys**	**2.41 ± 0.31** ^**ab**^	**2.55 ± 0.37** ^**ab**^	**2.66 ± 0.42** ^**ab**^	**3.06 ± 1.32** ^**a**^	**1.64 ± 0.16** ^**bc**^	**1.83 ± 0.21** ^**bc**^	**1.33 ± 0.28** ^**c**^	**1.95 ± 0.23** ^**bc**^
**Val**	**3.05 ± 0.75** ^**c**^	**5.41 ± 0.63** ^**a**^	**2.54 ± 0.09** ^**c**^	**5.05 ± 0.84** ^**ab**^	**0.73 ± 0.19** ^**d**^	**4.32 ± 0.31** ^**b**^	**0.92 ± 0.1** ^**d**^	**4.52 ± 0.39** ^**ab**^
**Met**	**0.25 ± 0.02** ^**c**^	**1.03 ± 0.08** ^**a**^	**0.65 ± 0.37** ^**b**^	**1.23 ± 0.16** ^**a**^	**0.53 ± 0.1** ^**b**^	**1.19 ± 0.2** ^**a**^	**0.62 ± 0.12** ^**b**^	**1.04 ± 0.1** ^**a**^
**Phe**	**0.92 ± 0.18** ^**d**^	**2.38 ± 0.02** ^**a**^	**1.08 ± 0.26** ^**d**^	**2.51 ± 0.34** ^**a**^	**0.44 ± 0.02** ^**e**^	**1.54 ± 0.17** ^**c**^	**0.47 ± 0.01** ^**e**^	**1.9 ± 0.21** ^**b**^
**Ile**	**0.5 ± 0.17** ^**b**^	**2.4 ± 0.11** ^**a**^	**0.64 ± 0.2** ^**b**^	**2.65 ± 0.34** ^**a**^	**0.6 ± 0.03** ^**b**^	**0.66 ± 0.19** ^**b**^	**0.5 ± 0.12** ^**b**^	**0.69 ± 0.03** ^**b**^
**Leu**	**3.92 ± 0.69** ^**b**^	**7.39 ± 0.21** ^**a**^	**2.79 ± 0.18** ^**bc**^	**6.29 ± 0.44** ^**a**^	**1.36 ± 0.37** ^**c**^	**0.68 ± 0.12** ^**c**^	**1.04 ± 0.32** ^**c**^	**5.58 ± 0.1** ^**b**^
**Lys**	**2.87 ± 0.47** ^**d**^	**9.41 ± 0.24** ^**a**^	**3.61 ± 0.12** ^**c**^	**8.46 ± 0.86** ^**b**^	**0.41 ± 0.18** ^**f**^	**10.08 ± 0.31** ^**a**^	**1.59 ± 0.18** ^**e**^	**8.07 ± 0.12** ^**b**^
**Trp**	**0.68 ± 0.31** ^**d**^	**1.66 ± 0.03** ^**a**^	**0.57 ± 0.1** ^**d**^	**1.35 ± 0.27** ^**ab**^	**0.54 ± 0.08** ^**d**^	**1.54 ± 0.47** ^**ab**^	**0.83 ± 0.07** ^** cd**^	**1.16 ± 0.17** ^**bc**^

**Note**: Asp: Aspartic acid, Glu: Glutamic acid, Ser: Serine, His: Histidine, Gly: Glycine, Thr: Threonine, Arg: Arginine, Ala: Alanine, Tyr: Tyrosine, Cys: Cystine, Val: Valine, Met: Methionine, Phe: Phenylalanine, Ile: Isoleucine, Leu: Leucine, Lys: Lysine, Trp: Tryptophan. Data reported in mg/g dried mycelia material. Statistical significance is indicated by different letters corresponding to *P* < 0.05 based on the Tukey′s test

### **Amino acid contents in *gcn4*-silenced strains under low-nitrogen concentrations**

Our previous study found that GCN4 was highly induced in response to a low concentration of nitrogen condition in *G. lucidum*. To assess the regulation of GCN4 on amino acid contents in *G. lucidum*, we examined the amino acid contents in *gcn4*-silenced strains. The content of almost every amino acid in the *gcn4*-silenced strains was significantly lower than that in the WT strain under the different nitrogen concentrations (Table 1). Silencing of *gcn4* resulted in a 54.2% decrease under the 60 mM Asn condition. However, under the 3 mM Asn condition, the contents of amino acids decreased by 59.8% and 62.3% in the *gcn4i-1* and *gcn4i-22* strains, respectively, compared with those in WT (Fig. 1A). Silencing of *gcn4* significantly decreased essential and tasteless amino acids under the 3 mM Asn (Fig. 1B, F). The contents of MSG-like taste amino acids decreased by 79.8% and 88.3% in the *gcn4i-1* and *gcn4i-22* strains compared with those in the WT but decreased by 69.8% and 68.3% under 60 mM Asn (Fig. 1C). The sweet amino acids in the *gcn4-*silenced strains showed no significant change compared with those in the WT under both conditions (Fig. 1D). In conclusion, GCN4 facilitated the accumulation of amino acids, especially the essential and MSG-like taste amino acids, under low-nitrogen conditions.

### The expression of amino acid metabolism-related genes under low-nitrogen concentrations

**Fig. 2:**
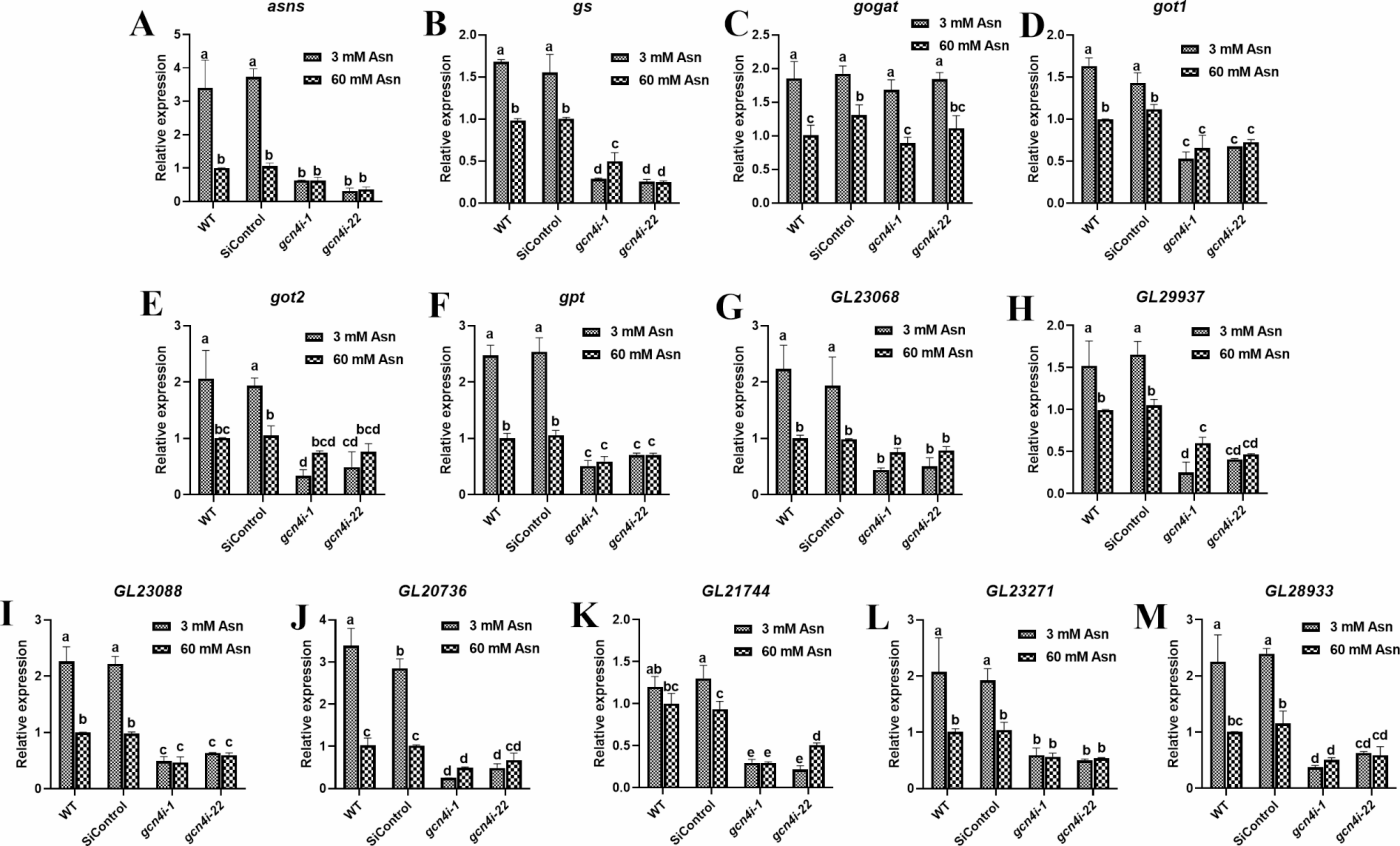
Involvement of GCN4 in regulating intracellular amino acid biosynthesis-related genes under low-nitrogen conditions. Relative expression of genes related to amino acid synthesis and transport in WT and *gcn4*-silenced strains under 3 mM and 60 mM Asn conditions. Expression of the asparagine synthetase gene (*asns*), glutamine synthetase gene (*gs*), glutamate synthase gene (*gogat*), glutamic oxaloacetic transaminase genes (*got1* and *got2*) and glutamic-pyruvic transaminase gene (*gpt*), as well as 7 that of amino acid transport genes (*Gl23068*, *Gl29937*, *Gl20736*, *Gl21744*, *Gl23271*, *Gl28933, Gl23088*), were determined. Expression levels of genes in the WT strain cultured under 60 mM Asn were defined as 1. The data are presented as the mean ± SD (n = 3). Statistical significance is represented by different letters corresponding to *P* < 0.05 based on Tukey′s test

Intracellular amino acids are obtained through many pathways, and GCN4 is one of the master regulators of genes involved in amino acid transport and biosynthesis [19]. We further investigated the expression of genes related to amino acid metabolism. Glutamic acid and aspartic acid are the most abundant amino acids in mushrooms, as well as in *G. lucidum* (Table 1). Therefore, the expression of genes including the asparagine synthetase gene (*asns*), glutamine synthetase gene (*gs*), glutamate synthase gene (*gogat*), glutamic oxaloacetic-transaminase gene (*got1* and *got2*) and glutamic-pyruvic transaminase gene (*gpt*) were detected. All these genes in the WT strain were significantly induced under 3 mM Asn, especially the *asns* gene, with 2.41-fold high expression than that under 60 mM Asn (Fig. 2A). Silencing of *gcn4* markedly inhibited the expression of almost all these genes, with a reduction of approximately 58.71-90.83% compared with WT under 3 mM Asn. Under 60 mM Asn, they decreased by 24.64-62.88% in *gcn4-*silenced strains compared with WT (Fig. 2A-F). However, the expression of *gogat* was unchanged in both the WT and in the *gcn4*-silenced strains (Fig. 2C). Furthermore, the expression of 7 amino acid transport genes (Gl23068, Gl29937, Gl20736, Gl21744, Gl23271, Gl28933, and Gl23088) was decreased by 71.29–92.59% in the *gcn4-*silenced strains under 3 mM Asn (Fig. 2G-M) and decreased by 22.84-60.1% under 60 mM Asn. These results indicated that silencing of *gcn4* decreased the expression of genes related to amino acid metabolism, especially under low-nitrogen conditions.

### **Effect of the TCA and glycolysis pathways in *gcn4*-silenced strains under low-nitrogen concentrations**

**Fig. 3:**
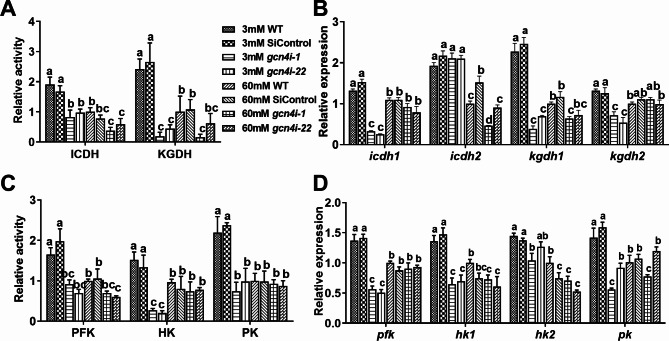
GCN4 is involved in regulating the TCA cycle and glycolysis under low-nitrogen conditions **(A)** Relative activities of key enzymes (ICDH and KGDH) associated with the TCA cycle in the WT and *gcn4*-silenced strains under 3 mM and 60 mM Asn **(B)** Relative expression of key enzyme synthesis genes in the TCA cycle of the WT and *gcn4*-silenced strains under 3 mM and 60 mM Asn. **(C)** Relative activities of key enzymes (PFK, HK and PK) associated with glycolysis in the WT and *gcn4*-silenced strains under 3 mM and 60 mM Asn. **(D)** Relative expression of key enzyme synthesis genes in glycolysis in the WT and *gcn4*-silenced strains under 3 mM and 60 mM Asn. *hk*: hexokinase. *pfk*: 6-phosphofructokinase. *pk*: pyruvate kinase. *cs*: citrate synthase. *icdh*: isocitrate dehydrogenase. *kgdh*: α-ketoglutarate dehydrogenase. The data are presented as the mean ± SD (n = 3). Statistical significance is represented by different letters according to Tukey′s test (*P* < 0.05)

Amino acids are also replenished through the TCA cycle and glycolysis pathway [31, 32]. Therefore, we further investigated the effect of GCN4 on the TCA cycle and glycolysis pathway. As shown in Fig. 3, compared with 60 mM Asn, the activities of mitochondrial isocitrate dehydrogenase (IDH) and α-ketoglutarate dehydrogenase (KGDH) in WT were significantly upregulated by 1.9 and 2.4-fold, respectively (Fig. 3A). The activities of hexokinase (HK), pyruvate kinase (PK), and phosphofructokinase (PFK) increased 1.65-, 1.52- and 2.2-fold, respectively, compared to 60 mM Asn (Fig. 3C). These results suggested that low-nitrogen conditions promote the TCA cycle and glycolysis pathway in *G. lucidum*.

Furthermore, under 3 mM Asn, after silencing *gcn4*, the enzyme activities of ICDH and KGDH decreased by 56% and 92.1%, respectively, compared with the WT strain. This treatment also led to 54.63%, 83.4% and 62.39% decreases in PFK, HK and PK activities, respectively. However, the activities of ICDH and three key enzymes involved in the glycolysis pathway showed no difference between the WT and *gcn4*-silenced strains under 60 mM Asn (Fig. 3A, B). The expression of genes in *gcn4*-silenced strains corresponded to the enzyme activities and exhibited a significant decrease of 35.38–83.15% under 3 mM Asn (Fig. 3B and D). The above results suggest that GCN4 enhances the TCA cycle and glycolysis pathway under low-nitrogen conditions.

### **The activity of TORC1 in *gcn4*-silenced strains under low-nitrogen concentrations**

**Fig. 4:**
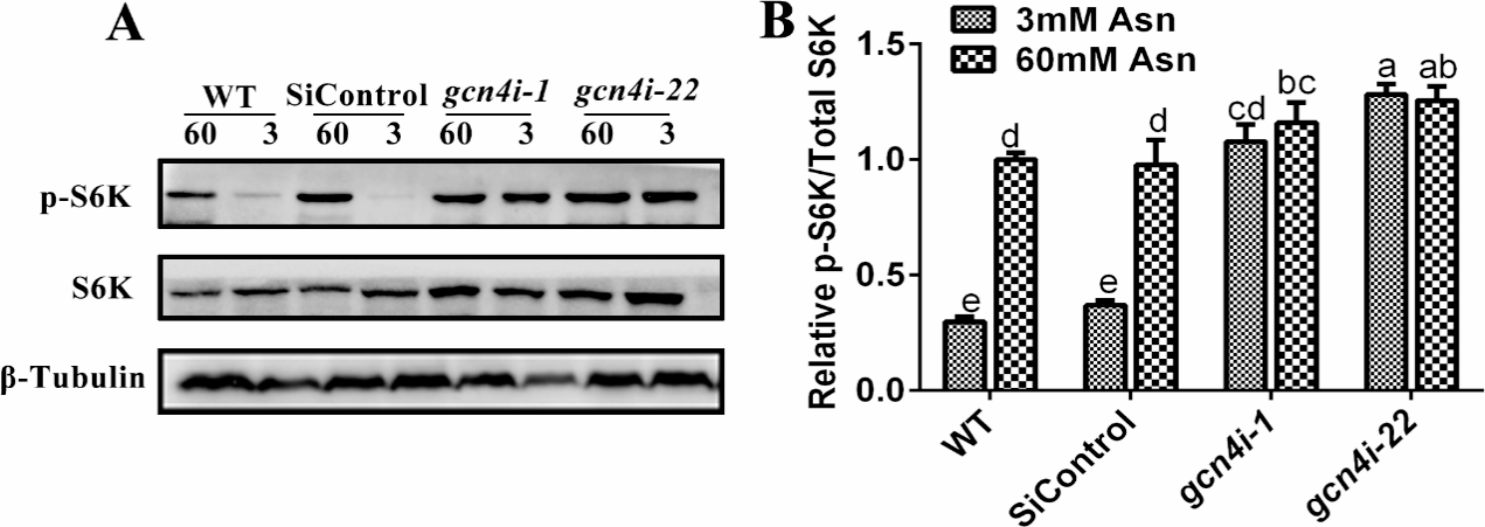
GCN4 inhibits TORC1 activity under low-nitrogen conditions. Western blot analysis of S6K phosphorylation levels in the WT and *gcn4*-silenced strains cultured under 3 mM and 60 mM Asn, with histograms showing the relative ratio of p-S6K/Total-S6K. The p-S6K/Total-S6K ratio in the WT strain grown under 60 mM Asn was defined as 1. The data are presented as the mean ± SD (n = 3). Statistical significance is represented by different letters according to Tukey′s test (*P* < 0.05)

The TORC1 plays a central role in sensing and regulating the metabolism and availability of amino acids [33]. To comprehensively analyse the regulation of amino acid homeostasis by GCN4, the activity of TORC1 (characterized by the phosphorylation state of S6K) was tested (Fig. 4A). The phosphorylation level of S6K in the WT strain significantly decreased by 66.4% at 3 mM Asn compared with 60 mM Asn. However, silencing of *gcn4* increased S6K phosphorylation, with 71.4% and 22% increases under 3 mM Asn and 60 mM Asn, respectively (Fig. 4B). This result suggests that GCN4 exerts an inhibitory effect on TORC1 activity, especially under low-nitrogen conditions.

## Discussion

**Fig. 5:**
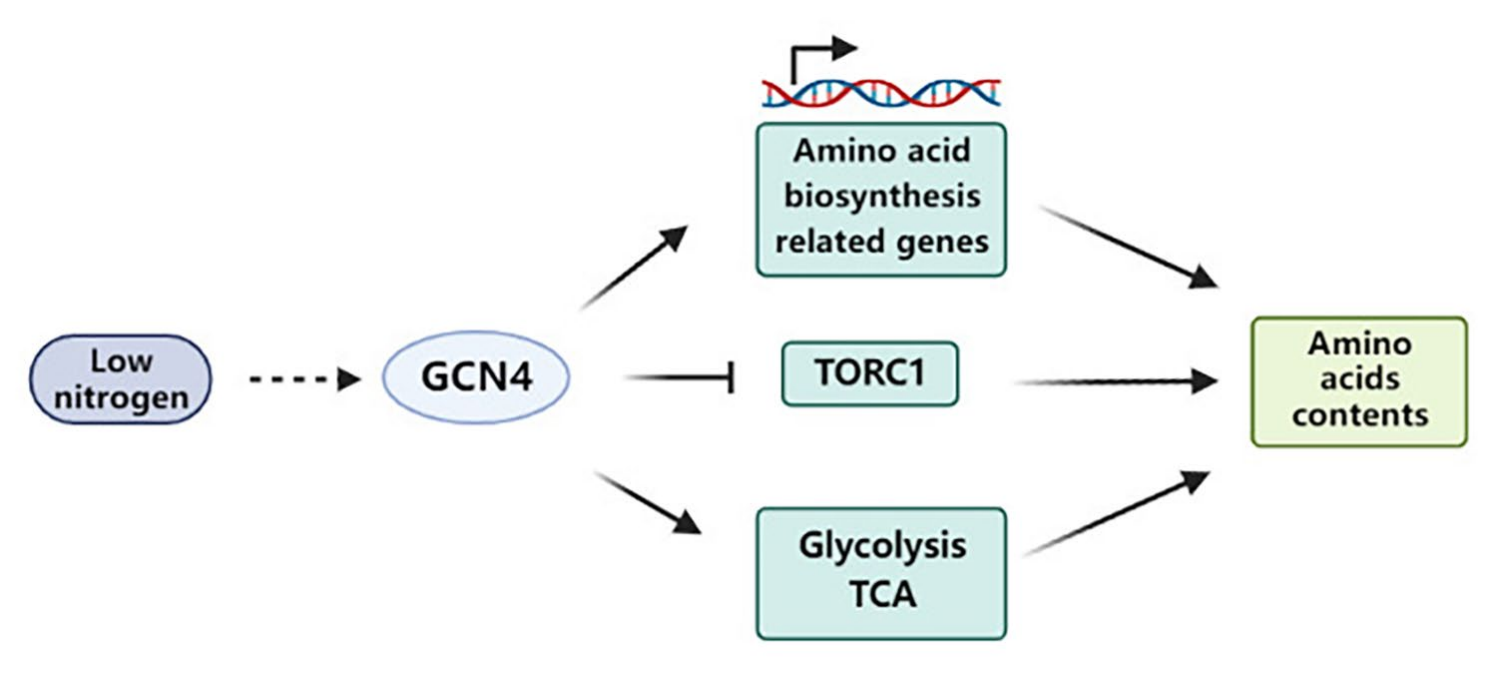
Schematic indicating that GCN4 regulates intracellular amino acid contents under low-nitrogen conditions. Under low-nitrogen conditions, GCN4 promotes the synthesis of intracellular amino acids through the transcriptional regulation of genes related to amino acid synthesis. In addition, GCN4 activates the TCA cycle and glycolytic pathway and inhibits the activity of TORC1 to replenish intracellular amino acids

Researchers have been committed to finding low-cost sources of protein that can replace those of animal origin [4]. Compared with plant or animal protein, edible mushroom production is faster and less expensive. In addition, mushrooms provide not only a significant protein content with a complete essential amino acid profile, but also the flavoring amino acids [2, 34]. Therefore, mushrooms protein can be used as a potential supplement to meat or plant proteins. Although the effects of environmental or cultivation conditions on the amino acid contents have been investigated, the regulation of amino acid metabolism in mushrooms is unclear. Our study provides new insight into the molecular mechanism of GCN4 in regulating amino acid metabolism in the mushroom *G. lucidum*. Under low-nitrogen conditions, GCN4 regulated the expression of genes involved in amino acid synthesis and transporter genes and promoted the glycolysis pathway and TCA cycle to provide precursors for amino acid biosynthesis. Additionally, the utilization of amino acids by TORC1 was hindered by GCN4, thereby maintaining the amino acid contents under low-nitrogen conditions in *G. lucidum* (Fig. 5). Taken together, the results of this study explain the regulatory mechanism of GCN4 in amino acid metabolism and provides the basis for further studies on the regulation of amino acid metabolism in edible mushrooms.

Nitrogen is a crucial nutrient for biological growth and development, playing a pivotal role in the synthesis of amino acids. In *Schizophyllum commune* and *Coprinus cinereus*, the mycelial growth and free amino acid concentration of mycelia grown on the nitrogen-rich medium (6.6 mM L-asparagine) increased compared with that on the nitrogen-poor medium (0.06 mM L-asparagine) [35, 36]. *G. lucidum* showed higher cell dry weight and ganoderic acids under organic nitrogen sources (glutamine, asparagine and glycine) [37]. Our study also found that using asparagine as the sole nitrogen source led to good growth and significantly activated GCN4 expression (Fig. S1). In addition, the amino acid contents under low-nitrogen conditions decreased by 62.96% compared with those under high-nitrogen conditions in *G. lucidum*. Low-nitrogen conditions activate a key transcription factor GCN4 to maintain mycelial growth and resistance to intracellular oxidative stress in *G. lucidum* [30]. Here, silencing of *gcn4* significantly reduced the intracellular amino acid contents and inhibited amino acid biosynthesis, which means that GCN4 contributes to maintaining intracellular amino acid homeostasis under low-nitrogen conditions in *G. lucidum*.

The functions of GCN4 in fungal growth and regulating genes involved in amino acid homeostasis are well known in some filamentous fungi and mammals [38–41]. In addition, the gene expression of specific key amino acid synthesis genes (ASNS, alanine aminotransferase 2, PSAT, serine hydroxymethyl transferase 2, pyrroline-5-carboxylate reductase and glutamate-oxaloacetate transaminase) genes in mammals was reported widely regulated by ATF4/GCN4 [19], Additionally, in yeast, the regulation of specific amino acid biosynthesis genes, including histidine and isoleucine/valine synthesis genes such as *his4*, *his3*, *ilv1*, and *ilv2* has also been observed [22]. Here, we examined the expression of genes involved in amino acid synthesis in *gcn4*-silenced. In *G. lucidum*, GCN4 enhanced the expression of *asns*, which is the typical target gene of GCN4 in mammals [41]. In addition, GOT coordinates carbon and nitrogen metabolism in organisms [42] and affects the intracellular glutamate content [43], which is the key metabolite in the intracellular amino acid biosynthesis [44]. In our study, transaminase genes (*got1* and *got2*) were regulated by GCN4. These results were consistent with previous studies showing that GCN4 is the master regulator of gene expression in amino acid synthesis in yeast [20]. Our study demonstrated for the first time in edible mushrooms that GCN4 regulated amino acid contents, as well as the expression of amino acid biosynthesis and transporter genes, which was important for improving the nutritional value of mushrooms.

The intermediate metabolites of carbon metabolism, such as pyruvate, oxaloacetate (OAA), and α-ketoglutarate (α-KG), are the respective precursors for the biosynthesis of amino acids [44, 45]. Here, we found that the activities of key enzymes and the expression of key genes in the TCA cycle and glycolysis were significantly upregulated when the mycelia were cultured under low-nitrogen conditions, which suggested that the TCA cycle and glycolysis might be activated to promote the synthesis of intracellular amino acids in response to nitrogen-limiting conditions. However, this upregulation disappeared in *gcn4*-silenced strains, suggesting that the GCN4 was involved in promoting the TCA cycle and glycolysis to provide precursors for amino acid synthesis and ultimately maintain intracellular amino acid homeostasis under nitrogen-limitation conditions. In addition, TORC1 is the central energy hub for sensing and regulating the metabolism and availability of amino acids [46]. Here, we found that TORC1 was also involved in the regulation of amino acids by GCN4. The phosphorylation of S6K was significantly reduced under nitrogen-limitation conditions, while silencing of *gcn4* activated phosphorylation of S6K, which is consistent with previous studies in yeast showing that the existence of GCN4 inhibited the phosphorylation of S6K [47]. Our results indicated that GCN4 was involved in the inhibition of TORC1 activity under low-nitrogen conditions. Considering that TORC1 recognizes and uses amino acids and activates S6K to regulate the synthesis of ribosomal proteins, as well as translationally regulated proteins [48], we hypothesized that GCN4 negatively regulates TORC1 under low-nitrogen conditions to inhibit protein synthesis, provide sufficient amino acids for cells, and ultimately comprehensively regulate amino acid homeostasis.

## Methods

### Fungal strains and culture conditions

The *G. lucidum* strain (ACCC53264) was obtained from the Agricultural Culture Collection of China (ACCC). The *gcn4*-silenced strains were obtained from our previous study [30]. The wild-type (WT) and *gcn4*-silenced strains of *G. lucidum* were cultivated in the complete yeast medium (CYM) which containing 2% glucose, 1% maltose, 0.46% KH_2_PO_4_, 0.2% yeast extract, 0.2% tryptone, and 0.05% MgSO_4_·7H_2_O. Cultivation under nitrogen limitation conditions was performed according to the method previously described. Cultures with different nitrogen sources were prepared by replacing the yeast extract and tryptone of CYM with 3 mM or 60 mM Asn [31, 33].

### Intracellular amino acid detection

The amino acid contents were determined using the O-phthalaldehyde (OPA) precolumn derivatization method [49]. Mycelial samples (0.03 g) were ground and added to 500 µL of NaOH-sodium borate buffer (pH 9.5) for ultrasonic extraction for 30 min. Then, 200 µL of supernatant was mixed with 100 µL of OPA reagent solution (30 mM OPA, 70 mM 2-mercaptoethanol, 50 mM sodium borate, pH 9.5) for 1 min. Next, 200 µL of 100 mM KH_2_PO_4_ buffer was added to terminate the reaction. The supernatant was analysed using a UPLC (Agilent Technologies, Santa Clara, CA, USA) equipped with a C_18_ column (1.8 μm, 50 mm × 2.1 mm I.D.). A gradient elution was performed with 20 mM aqueous acetic acid (mobile phase A, 85–20%) and 20 mM aqueous acetic acid/methanol/acetonitrile (1:2:2, v/v; mobile phase B, 15–80%). A fluorescence detector was used to monitor fluorescence at excitation and emission wavelengths of 340 and 455 nm, respectively.

### qRT‒PCR analysis

The strains cultivated at 28 °C were collected for the total RNA extraction using RNAiso Plus reagent (TaKaRa, Dalian, China). cDNA was obtained by reverse transcription using a 5x All-In-One RT MasterMix kit (Vazyme, Nanjing, China). qRT‒PCR was performed using EvaGreen 2X qPCR MasterMix (Vazyme, Nanjing, China). Gene expression was normalized against the 18 S reference gene using the primers listed in Table S1.

### Enzymatic activity assays

The enzyme activities of mitochondrial isocitrate dehydrogenase (IDH), α-ketoglutarate dehydrogenase (KGDH), hexokinase (HK), pyruvate kinase (PK), and phosphofructokinase (PFK) were analyzed using detection kit (Solarbio, Beijing, China), respectively, according to the manufacturers’ protocols. All values were normalized to the total protein levels. The protein content was quantified by the Bradford Protein Assay Kit (Sangon, Shanghai, China). All these enzymatic activities in the control group were normalized to 1.0.

### Western blotting assays

Western blotting was conducted according to a previous method using polyclonal antibodies against GCN4 (1:1,000) and anti-total p70 S6K, anti-phospho (T389)-S6K, and β-tubulin antibodies (9202, 9205, and 2146, 1:2,000, Cell Signaling Technology, Shanghai, China). HRP-conjugated goat anti-rabbit antibody was used as secondary antibody [33].

### Statistical analysis

Statistical analysis in this study was performed using GraphPad Prism 6 (GraphPad Software, San Diego, USA). The data from at least three independent sample measurements were averaged, and the values shown are the means and standard deviations (SDs). Data analyses using Student’s *t* test were used for two-sample comparisons, and one-way analysis of variance (ANOVA) with Duncan’s posttest was used for multiple comparisons.

### Electronic supplementary material

Below is the link to the electronic supplementary material.

Supplementary Material 1

Supplementary Material 2

## Data Availability

All data generated or analyzed during this study are included in this published article.

## References

[1] Strong PJ, Self R, Allikian K, Szewczyk E, Speight R, O’Hara I, Harrison MD (2022). Filamentous fungi for future functional food and feed. Curr Opin Biotechnol.

[2] Bach F, Helm CV, Bellettini MB, Maciel GM, Haminiuk CWI (2017). Edible mushrooms: a potential source of essential amino acids, glucans and minerals. Int J Food Sci Technol.

[3] Humpenoder F, Bodirsky BL, Weindl I, Lotze-Campen H, Linder T, Popp A (2022). Projected environmental benefits of replacing beef with microbial protein. Nature.

[4] Gonzalez A, Cruz M, Losoya C, Nobre C, Loredo A, Rodriguez R, Contreras J, Belmares R (2020). Edible mushrooms as a novel protein source for functional foods. Food Funct.

[5] Mau JL, Lin HC, Ma JT, Song SF (2001). Non-volatile taste components of several speciality mushrooms. Food Chem.

[6] Xu J, Xu D, Hu Q, Ma N, Pei F, Su A, Ma G (2022). Immune regulatory functions of biologically active proteins from edible fungi. Front Immunol.

[7] Xu X, Yan H, Chen J, Zhang X (2011). Bioactive proteins from mushrooms. Biotechnol Adv.

[8] Kivrak I, Kivrak S, Harmandar M (2014). Free amino acid profiling in the giant puffball mushroom (Calvatia gigantea) using UPLC-MS/MS. Food Chem.

[9] Liu F, Wang W, Chen BZ, Xie BG (2015). Homocitrate Synthase expression and lysine content in fruiting body of different developmental stages in Flammulina velutipes. Curr Microbiol.

[10] Chen CC, Ho CT (1986). Identification of sulfurous compounds of shiitake mushroom (Lentinus edodes sing). J Agric Food Chem.

[11] Yamaguchi S. The umami taste. In *Food taste chemistry* Edited by Boudreau JC. Washington, DC: American Chemical Society; 1979: 33–51.[Boudreau JC (Series Editor)].

[12] Rotzoll N, Dunkel A, Hofmann T (2006). Quantitative studies, taste reconstitution, and omission experiments on the key taste compounds in morel mushrooms (Morchella deliciosa fr). J Agric Food Chem.

[13] Lavelli V, Proserpio C, Gallotti F, Laureati M, Pagliarini E (2018). Circular reuse of bio-resources: the role of Pleurotus spp. in the development of functional foods. Food Funct.

[14] Gupta A, Sharma S, Saha S, Walia S (2013). Yield and nutritional content of Pleurotus sajor caju on wheat straw supplemented with raw and detoxified mahua cake. Food Chem.

[15] Li W, Li XB, Yang Y, Zhou F, Liu YF, Zhou S (2015). Effects of different carbon sources and C/N values on nonvolatile taste components of Pleurotus eryngii. Int J Food Sci Technol.

[16] Tsai SY, Huang EW, Lin CP (2017). Compositional differences of the winter culinary-medicinal mushroom, *Flammulina velutipes* (*Agaricomycetes*), under three types of light conditions. Int J Med Mushrooms.

[17] Liu J, Li Q, Jiang P, Xu Z, Zhang D, Zhang L (2019). Overexpression of the saccharopine dehydrogenase gene improves lysine biosynthesis in Flammulina velutipes. J Basic Microbiol.

[18] Liu Y, Lei XY, Chen LF, Bian YB, Yang H, Ibrahim SA, et al. A novel cysteine desulfurase influencing organosulfur compounds in *Lentinula edodes*. Sci Rep. 2015;5. 10.1038/srep10047 PMC4460571 26054293

[19] Bröer S, Bröer A (2017). Amino acid homeostasis and signalling in mammalian cells and organisms. Biochem J.

[20] Natarajan K, Meyer MR, Jackson BM, Slade D, Roberts C, Hinnebusch AG, Marton MJ (2001). Transcriptional profiling shows that Gcn4p is a master regulator of gene expression during amino acid starvation in yeast. Mol Cell Biol.

[21] Hinnebusch AG (2005). Translational regulation of GCN4 and the general amino acid control of yeast. Annu Rev Microbiol.

[22] Hinnebusch AG, Natarajan K (2002). Gcn4p, a master regulator of gene expression, is controlled at multiple levels by diverse signals of starvation and stress. Eukaryot Cell.

[23] Arndt K, Fink GR (1986). GCN4 protein, a positive transcription factor in yeast, binds general control promoters at all 5’ TGACTC3’ sequences. Proc Natl Acad Sci.

[24] Krappmann S, Bignell Elaine M, Reichard U, Rogers T, Haynes K, Braus Gerhard H (2004). The *aspergillus fumigatus* transcriptional activator CpcA contributes significantly to the virulence of this fungal pathogen. Mol Microbiol.

[25] Eckert SE, Kubler E, Hoffmann B, Braus GH (2000). The tryptophan synthase-encoding trpB gene of aspergillus nidulans is regulated by the cross-pathway control system. Mol Gen Genetics: MGG.

[26] Goc A, Weglenski P (1988). Regulatory region of the aspergillus nidulans argB gene. Curr Genet.

[27] Bishop KS, Kao CH, Xu Y, Glucina MP, Paterson RR, Ferguson LR (2015). From 2000years of Ganoderma lucidum to recent developments in nutraceuticals. Phytochemistry.

[28] Chen S, Xu J, Liu C, Zhu Y, Nelson DR, Zhou S, Li C, Wang L, Guo X, Sun Y (2012). Genome sequence of the model medicinal mushroom *Ganoderma lucidum*. Nat Commun.

[29] Shi L, Fang X, Li M, Mu D, Ren A, Tan Q (2012). Development of a simple and efficient transformation system for the basidiomycetous medicinal fungus Ganoderma lucidum. World J Microbiol Biotechnol.

[30] Lian LD, Wang LS, Song SQ, Zhu J, Liu R, Shi L, et al. GCN4 regulates secondary metabolism through activation of antioxidant gene expression under nitrogen limitation conditions in *Ganoderma lucidum*. Appl Environ Microbiol. 2021;87:e0015621. 10.1128/AEM.00156-21 PMC8231712 33962980

[31] Ye J, Mancuso A, Tong X, Ward PS, Fan J, Rabinowitz JD, Thompson CB (2012). Pyruvate kinase M2 promotes de novo serine synthesis to sustain mTORC1 activity and cell proliferation. Proc Natl Acad Sci U S A.

[32] Kurmi K, Haigis MC (2020). Nitrogen metabolism in cancer and immunity. Trends Cell Biol.

[33] Chi H (2012). Regulation and function of mTOR signalling in T cell fate decisions. Nat Rev Immunol.

[34] Zhang Y, Venkitasamy C, Pan ZL, Wang W (2013). Recent developments on umami ingredients of edible mushrooms - a review. Trends Food Sci Technol.

[35] Hanks JN, Hearnes JM, Gathman AC, Lilly WW (2003). Nitrogen starvation-induced changes in amino acid and free ammonium pools in Schizophyllum commune colonies. Curr Microbiol.

[36] Ulrich CE, Gathman AC, Lilly WW (2007). Amino acid pool composition of the basidiomycete Coprinus cinereus. Can J Microbiol.

[37] Zhao W, Xu JW, Zhong JJ (2011). Enhanced production of ganoderic acids in static liquid culture of *Ganoderma lucidum* under nitrogen-limiting conditions. Bioresour Technol.

[38] Tripathi G, Wiltshire C, Macaskill S, Tournu H, Budge S, Brown AJP (2002). Gcn4 co-ordinates morphogenetic and metabolic responses to amino acid starvation in *Candida albicans*. EMBO J.

[39] Ebbole DJ, Paluh JL, Plamann M, Sachs MS, Yanofsky C (1991). cpc-1, the general regulatory gene for genes of amino acid biosynthesis in Neurospora crassa, is differentially expressed during the asexual life cycle. Mol Cell Biol.

[40] Hope IA, Struhl K (1985). GCN4 protein, synthesize in vitro, binds HIS3 regulatory sequences: implications for general control of amino acid biosynthetic genes in yeast. Cell.

[41] Siu F, Bain PJ, LeBlanc-Chaffin R, Chen H, Kilberg MS (2002). ATF4 is a mediator of the nutrient-sensing response pathway that activates the human asparagine synthetase gene. J Biol Chem.

[42] Toney MD (2014). Aspartate aminotransferase: an old dog teaches new tricks. Arch Biochem Biophys.

[43] Liu H, Qiao J, Shangguan J, Zhu J (2023). Effects of glutamate oxaloacetate transaminase on reactive oxygen species in Ganoderma lucidum. Appl Microbiol Biotechnol.

[44] Bianchi F, Van’t Klooster JS, Ruiz SJ, Poolman B. Regulation of amino acid transport in *Saccharomyces cerevisiae*. Microbiol Mol Biol Rev. 2019;83. 10.1128/MMBR.00024-19 PMC7405077 31619504

[45] Yang X, Xia R, Yue C, Zhai W, Du W, Yang Q, Cao H, Chen X, Obando D, Zhu Y (2018). ATF4 regulates CD4 T cell immune responses through metabolic reprogramming. Cell Rep.

[46] Mossmann D, Park S, Hall MN (2018). mTOR signalling and cellular metabolism are mutual determinants in cancer. Nat Rev Cancer.

[47] Staschke KA, Dey S, Zaborske JM, Palam LR, McClintick JN, Pan T, Edenberg HJ, Wek RC (2010). Integration of general amino acid control and target of rapamycin (TOR) regulatory pathways in nitrogen assimilation in yeast. J Biol Chem.

[48] González A, Hall MN (2017). Nutrient sensing and TOR signaling in yeast and mammals. EMBO J.

[49] Dai Z, Wu Z, Jia S, Wu G (2014). Analysis of amino acid composition in proteins of animal tissues and foods as pre-column o-phthaldialdehyde derivatives by HPLC with fluorescence detection. J Chromatogr B.

