# Supplementary material for: The transcription factor GCN4 contributes to maintaining intracellular amino acid contents under nitrogen-limiting conditions in the mushroom Ganoderma lucidum
Source: Microb Cell Fact. 2023 Oct 10;22:205. doi: 10.1186/s12934-023-02213-z (PMC10563202; doi:10.1186/s12934-023-02213-z)
Supplement: Supplementary file 1 — Supplementary Material 1 [file 12934_2023_2213_MOESM1_ESM.docx]

**Table S1** Oligonucleotide primers used this article

| Primer | Sequence (5′to 3′) | Description |
| --- | --- | --- |
| RT-18S-F | TATCGAGTTCTGACTGGGTTGT | Detects the 18S expression |
| RT-18S-R | ATCCGTTGCTGAAAGTTGTAT |  |
| RT-ASNS-F | CTCGCCATTGTCGGTGTT | Detects the *asns* expression |
| RT-ASNS-R | GGTTATAGATTTCGCCGTTCA |  |
| RT-GOT1-F | TTGGTGTTACCGAGGCGTTT | Detects the *got1* expression |
| RT-GOT1-R | AGGTACTCCTTGTCCGGGTT |  |
| RT-GOT2-F | GTCGTCAAGAAGGCGACTGA | Detects the *got2* expression |
| RT-GOT2-R | AACTCGGGGAGACCAGTGAT |  |
| RT-GPT-F | CAGAAGGGACTCGACCAACC | Detects the *gpt* expression |
| RT-GPT-R | CTGAGGCCATTTTCCCCGTA |  |
| RT-GL29973-F | CTTCAATACACGCCCTTGGC | Detects the aa transporter expression |
| RT-GL29973-R | TGCAGGACACATAGGCGAAA |  |
| RT-GL23068-F | ATCCTCGCATGCGCTATCAA | Detects the aa transporter expression |
| RT-GL23068-R | GTCGATGACAAGACCGGTGA |  |
| RT-GL21744-F | GCTGGTCTCTTGCTGACCTT | Detects the aa transporter expression |
| RT-GL21744-R | CCGACTCTCCAAACCAACGA |  |
| RT-GL23088-F | TACGAACGCTATCTCCGTGC | Detects the aa transporter expression |
| RT-GL23088-R | GCTGTGTATGCCGCTTGATG |  |
| RT-GL20736-F | GCTTCGTCTCTCCTGCACTT | Detects the aa transporter expression |
| RT-GL20736-R | TGTTGGGTCCCCAATACTGC |  |
| RT-GL28933-F | TTCTTCTCCCCCTCCTACGG | Detects the aa transporter expression |
| RT-GL28933-R | TGACCCATGTGTGCCAAGTT |  |
| RT-GL23271-F | CGACCGTTAACTGCTTCCCT | Detects the aa transporter expression |
| RT-GL23271-R | CGTGAGACGCTCCAAGATCA |  |
| RT-GL26783-F | CCAGTTCGTCCTAAAGCA | Detects the *hk1* expression |
| RT-GL26783-R | CAGAAGCAAAGTCCGTGA |  |
| RT-GL20491-F | AGGAACAGAAGCAGGATGA | Detects the *hk2* expression |
| RT-GL20491-R | CCTTTAGGGATAAGACCAGAC |  |
| RT-GL26108-F | GACTTGGATCGTCCGTGAG | Detects the *pfk* expression |
| RT-GL26108-R | GGGCTATTGCCATTCTTCT |  |
| RT-GL30680-F | TGTGCTGAAACAAACTGCC | Detects the *pk* expression |
| RT-GL30680-R | AGAGCCTGCTGAACCAA |  |
| RT-GL20627-F | CGATTCGTGGGTTTGCC | Detects the *icdh1* expression |
| RT-GL20627-R | CGGTGCTCGTAATAGATGC |  |
| RT-GL31424-F | TCAACACCGTCCTCATCC | Detects the *icdh2* expression |
| RT-GL31424-R | CTCAGAAGCGTCCCAAGT |  |
| RT-GL22262-F | GCTCGCTGCTGACTGGC | Detects the *kgdh1* expression |
| RT-GL22262-R | ATGCGTGGCTGCGTGAA |  |
| RT-GL15965-F | CACGCAGGCGATGGAAA | Detects the *kgdh2* expression |
| RT-GL15965-R | AGCGGGACGACGACACC |  |
