# Supplementary figures and images for: The transcription factor GCN4 contributes to maintaining intracellular amino acid contents under nitrogen-limiting conditions in the mushroom Ganoderma lucidum
Source: Microb Cell Fact. 2023 Oct 10;22:205. doi: 10.1186/s12934-023-02213-z (PMC10563202; doi:10.1186/s12934-023-02213-z)

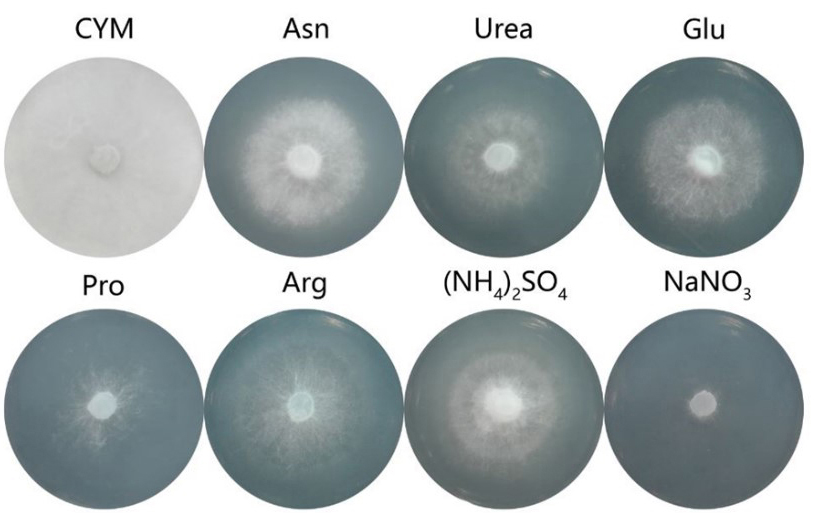

Supplement: Supplementary file 2 — Supplementary Material 2 [file 12934_2023_2213_MOESM2_ESM.jpg]
